# A Rare Prostatic Diagnosis of an Old Man: A Pure Prostatic Leiomyoma

**DOI:** 10.1155/2013/741235

**Published:** 2013-08-24

**Authors:** W. M. van Ulden-Bleumink, P. G. B. Dom, B. P. M. Ramakers, N. P. H. van Adrichem

**Affiliations:** ^1^Department of Urology, Meander Medisch Centrum, Postbus 1502, 3800 BM Amersfoort, The Netherlands; ^2^Department of Clinical Pathology, Meander Medisch Centrum, Postbus 1502, 3800 BM Amersfoort, The Netherlands

## Abstract

A pure leiomyoma of the prostate is a rare benign tumor. An 82-year-old man was referred to our urology department with gross hematuria and complete urinary retention. Examination revealed a benign prostatic hyperplasia. Transrectal ultrasound showed a prostate of 125 mL. Serum PSA was 1.9 *µ*g/L. A simple retropubic prostatectomy was performed. Histopathological examination showed a pure leiomyoma of the prostate, without the presence of glandular prostate tissue. The diagnosis, characteristics, and treatment of this tumor are described.

## 1. Background

A leiomyoma of the prostate is a relatively rare benign tumor of the prostate of smooth muscle origin. Most cases are diagnosed at histopathological examination after prostate biopsies or a resection of the prostate. We present a patient in whom the entire specimen, after retropubic prostatectomy, consisted of smooth muscle, a pure leiomyoma. 

## 2. Case Presentation

An 82-year-old man presented at the urology department with intermittent painless gross hematuria and LUTS. His previous medical history was diabetes mellitus type II, stroke, and myocardial infarction with a CABG and use of coumarin. On digital rectal examination, a smooth, firm, and elastic enlarged prostate was palpated. Laboratory tests showed a serum PSA of 1.9 *μ*g/L. Urethrocystoscopy, a CT intravenous pyelogram, and urinalysis revealed no abnormalities. The haematuria was referred to as a prostatic bleeding to benign prostatic hyperplasia. Tamsulosin and dutasteride were started, which improved urination, and hematuria no longer occurred. One year later, the patient presented with acute urinary retention and received a catheter. The urodynamic study showed infravesical obstruction with a normal detrusor contraction. Transrectal ultrasound showed a gland of 125 mL, with no abnormalities. A straightforward simple retropubic prostatectomy was performed. Postoperatively postvoid residual was low, and the patient was discharged. After restarting his coumarin, gross haematuria with clot retention occurred, for which hospitalization and conservative treatment were followed.

**Figure 1:**
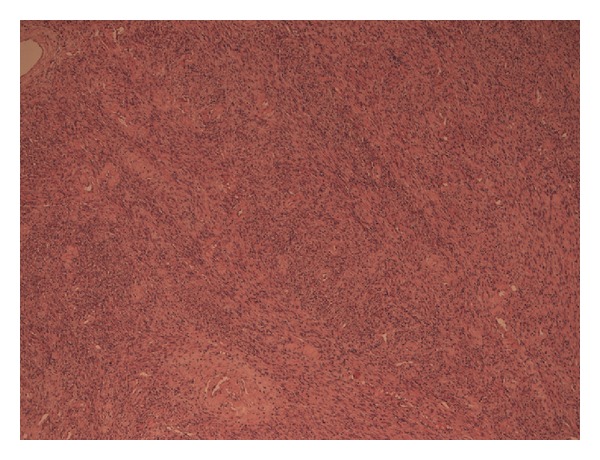
Cell image of a prostatic leiomyoma, characterized by arranged intersecting bundles not atypical smooth muscle cells.

**Figure 2:**
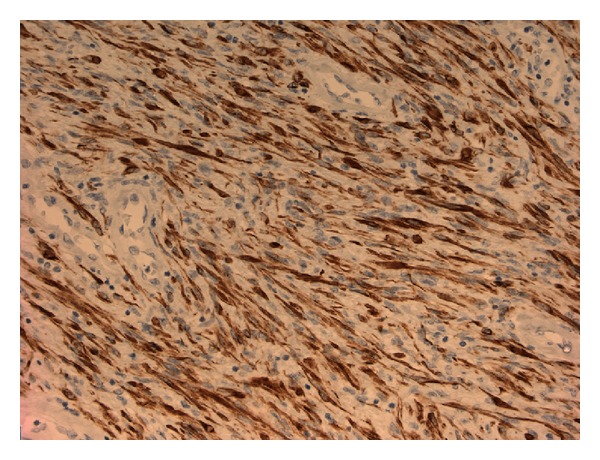
The spindle cell population shows desmin positivity.

**Figure 3:**
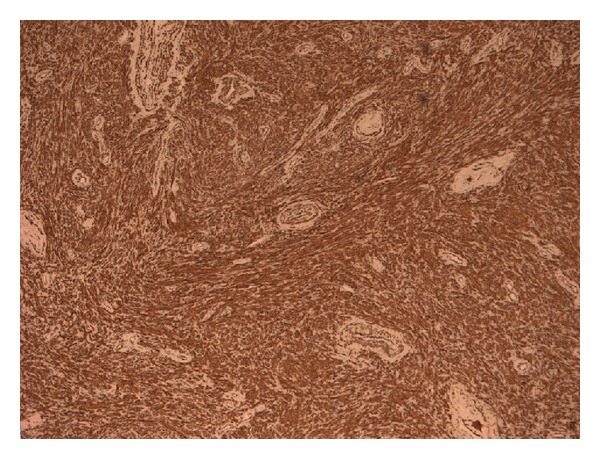
The spindle cell population shows strong SMA positivity.

Histopathological examination showed a spindle cell tumor with no atypical smooth muscle cells (Figure 1), and the cells were positive for desmin and smooth muscle actin (SMA) (Figures 2 and 3). There was no evidence of malignancy, and no glandular prostatic tissue was found, so the diagnosis was a pure leiomyoma of the prostate.

## 3. Discussion

A leiomyoma of the prostate is a rare pathologic finding, especially when no glandular prostatic tissue is present. Therefore, we call it a pure leiomyoma [1, 2]. The first case report of a leiomyoma of the prostate—found at autopsy—was published in 1876. It described a leiomyoma of the prostate as a tumor of smooth muscle similar to smooth muscle tumors of the uterus [3]. Several decades passed until Kaufman and Berneike in 1951 defined the pure leiomyoma of the prostate [4]. Since then, the international literature published about 15 case reports concerning a pure prostatic leiomyoma. Unlike our case where an old man presented with gross haematuria, other reports describe middle-aged men presented with obstructive urinary or acute urinary retention [1, 3, 5–7].

A pure leiomyoma is usually found by histopathological examination in prostate biopsies or after a resection of the prostate. There are different types of spindle cell tumors of the prostate: both the leiomyoma and the stromal tumors contain smooth muscle tissue. Macroscopically, a leiomyoma relates to a well-defined nodular formation [5]. Microscopically, it is characterized by intersecting bundles not atypical, smooth muscle cells with uniform cigar-shaped and slightly vesicular nuclei. The spindle cell population is desmin, smooth muscle actin (SMA) and androgen receptor positive [8]. If there is also an increased mitotic activity and necrosis, it is a leiomyosarcoma.

Considering the pathogenesis of leiomyoma, several theories describe it as a result of a chronic inflammatory reaction or embryological ability [4]. A leiomyoma can develop in all organs containing smooth muscle; most common places are the uterus and gastrointestinal tract [9].

## 4. Conclusion

A pure leiomyoma of the prostate is a rare, benign disorder with complete absence of glandular prostatic tissue, diagnosed by histopathological examination. This paper shows that, in case of a pure leiomyoma, presentation can be also with gross haematuria, and a simple retropubic prostatectomy is an appropriate treatment for an elderly man.
